# Contribution of BH3-domain and Transmembrane-domain to the Activity and Interaction of the Pore-forming Bcl-2 Proteins Bok, Bak, and Bax

**DOI:** 10.1038/s41598-018-30603-6

**Published:** 2018-08-20

**Authors:** Daniel Stehle, Melanie Grimm, Stephanie Einsele-Scholz, Friederike Ladwig, Janina Johänning, Gerd Fischer, Bernhard Gillissen, Klaus Schulze-Osthoff, Frank Essmann

**Affiliations:** 1 Department of Molecular Medicine, Interfaculty Institute for Biochemistry, University of Tübingen, 72076 Tübingen, Germany; 2 Department of Plant Physiology, Center for Plant Molecular Biology (ZMBP), University of Tübingen, 72076 Tübingen, Germany; 3 Dr. Margarete Fischer-Bosch Institute for Clinical Pharmacology (IKP), 70376 Stuttgart, Germany; 4 Clinical and Molecular Oncology, University Medical Center Charité, 13125 Berlin, Germany; 5 German Cancer Consortium (DKTK) and German Cancer Research Center (DKFZ), 69120 Heidelberg, Germany

## Abstract

Central to intrinsic apoptosis signaling is the release of cytochrome c from mitochondria, which depends on the pro-apoptotic effector proteins Bax, Bak or Bok. These pore-forming effector proteins share four Bcl-2 homology (BH) domains, a functionally essential and conserved sequence of hydrophobic amino acids in their BH3-domain and a C-terminal transmembrane-domain whose specific function remains rather unknown. To elucidate the molecular basis of Bok-mediated apoptosis we analyzed apoptosis induction by transmembrane-domain deficient BokΔTM compared to the respective Bax and Bak proteins and proteins in which the first leucine in the BH3-stretch was mutated to glutamic acid. We show that deletion of the C-terminal transmembrane-domain reduces the pro-apoptotic function of each protein. Mutation of the first leucine in the BH3-domain (L78E) blocks activity of Bak, while mutation of the homologue residues in Bax or Bok (L63E and L70E respectively) does not affect apoptosis induction. Unexpectedly, combined mutation of the BH3-domain and deletion of the transmembrane-domain enhances the pro-apoptotic activity of Bok(L70E)ΔTM by abolishing the interaction with anti-apoptotic proteins, especially the primary Bok-inhibitory protein Mcl-1. These results therefore suggest a specific contribution of the transmembrane-domain to the pro-apoptotic function and interaction of Bok.

## Introduction

The pro-apoptotic multidomain proteins (MDPs) of the Bcl-2 family, i.e. Bax, Bak, and Bok^1^, mediate the release of cytochrome c from the mitochondrial intermembrane space into the cytosol. Only recently it was shown that Bax mediates cytochrome c release by forming rings in the outer mitochondrial membrane^2,3^. These rings, however, are different from the prominent higher order molecular clusters formed by active Bax and Bak at the mitochondria^4,5^. Detailed structural analyses propose a multi-step model for the oligomerization of pro-apoptotic effector MDPs, in which activator BH3-only proteins induce a conformational change in MDPs, e.g. Bax, that initiates their translocation to mitochondria and insertion into the outer membrane of mitochondria. At the mitochondria anti-apoptotic Bcl-2 proteins mediate retrotranslocation of MDPs back to the cytosol. Although each MDP individually and independently induces cytochrome c release^6^, the underlying molecular mechanisms and even their oligomeric structures potentially differ significantly. Especially the facts that inactive Bak is constitutively localized at mitochondrial membranes whereas inactive Bax is a soluble monomer indicates prominent rather than subtle molecular differences^7^.

In their inactive conformation the N-terminus of Bax and Bak folds back onto the globular protein^8^. Upon activation Bax and Bak change their conformation and commonly expose their N-terminus, an event that can be detected by conformation specific antibodies^9,10^. An additional conformational change (in Bax) exposes the C-terminal transmembrane-domain and mediates binding to the mitochondrial outer membrane – where Bak is already localized. For Bok only superficial studies of the molecular basis for its pro-apoptotic function have been made. It has been shown that overexpressed EGFP-Bok forms oligomers at the mitochondrial outer membrane^11^. Recently, Fernandez-Marrero *et al*.^12^ published the formation of oligomeric Bok^ΔC^ (aa 1–189, lacking the transmembrane-domain) structures in large and giant unilamellar vesicles (LUVs and GUVs). Notably, pore formation in LUVs and GUVs was shown for recombinant Bok^ΔC^ lacking the transmembrane-domain. Strikingly, however, permeabilization of mitochondria from Bax^−/−^/Bak^−/−^ MEFs was not achieved by Bok^ΔC^ ^12^.

Llambi *et al*. followed up the previously proposed role of Bok in ER-stress dependent apoptosis^13,14^. They confirm that Bok induces apoptosis in the absence of Bax and Bak in response to ER stress^14^. Furthermore, Llambi *et al*. claim that Bok is constitutively active and degraded by the proteasome. Upon ER-stress stabilization of Bok is induced which promotes oligomerization of Bok and apoptosis^15^. The proteasomal degradation proposed for the regulation of Bok activity significantly differs from the mechanism of Bax and Bak activation that is controlled by interaction with anti-apoptotic Bcl-2 and/or BH3-only proteins that regulate induction of a conformational change. The interaction with anti-apoptotic proteins is another aspect of incongruent data regarding Bok. Initially Bok was found in a two-hybrid screen to interact with Mcl-1 and A1^11^. Later Echeverry *et al*.^14^ found that Mcl-1 knockout reduces survival of MEFs upon 4-hydroxy-tamoxifen (4-OHT) induced expression of Bok while Bok and Mcl-1 did not co-immunoprecipitate. Recently, Llambi *et al*. showed that Mcl-1 co-immunoprecipitates Bok and delays Bok mediated LUV permeabilization but the induction of cell death by Bok was not inhibited by Mcl-1. Interestingly, the yet-to-be verified Bok antagonist Mcl-1 itself is quickly degraded by the proteasome with a half-life of 0.5–3 h^16,17^ – the same regulatory mechanism Llambi *et al*. propose for Bok^15^. These findings raise questions about the function of Bok as a genuine MDP, the regulation of Bok-induced apoptosis, if Bok interacts with Mcl-1 and whether Bok acts independently of Bax and Bak or not^6,14,15^.

To answer some of these questions we performed overexpression experiments in different cellular systems that are commonly used to investigate the function of Bcl-2 family proteins, including HCT116/WT and Bax/Bak deficient HCT116 cells (HCT116/DKO)^18^. We also investigated apoptosis induction by Bok compared to Bax and Bak in the presence of co-expressed anti-apoptotic proteins, most importantly the proposed antagonist Mcl-1. Our analyses in HCT116/DKO confirm that Bok-induced apoptosis is independent of Bax and Bak and can be blocked by Mcl-1. To gain a systematic picture of the molecular basis for MDP mediated apoptosis we investigated apoptosis induction by transmembrane-domain deficient variants (ΔTM) of the MDPs and mutant variants in which the first conserved amino acid residue (leucine) of the hydrophobic BH3-stretch was substituted by a glutamic acid (L70E). These analyses indicate that the regulation of Bok activity differs from that of Bak and presumably Bax because mutation of the BH3-domain does not influence apoptosis induction by Bok. Unexpectedly the L70E mutation of the BH3-domain restores apoptosis induction by Bok lacking the TM-domain. We show that the enhanced apoptosis induction by Bok(L70E)ΔTM is caused by abrogated transmembrane-dependent interaction with Mcl-1 in combination with TM-domain independent localization of Bok to the mitochondrial membrane^12^. In short, the transmembrane-domain of Bok is dispensable for apoptosis induction but essential for efficient inhibition of Bok-induced apoptosis by Mcl-1.

## Results

### Apoptosis induction by Bax, Bak, and Bok

**Figure 1:**
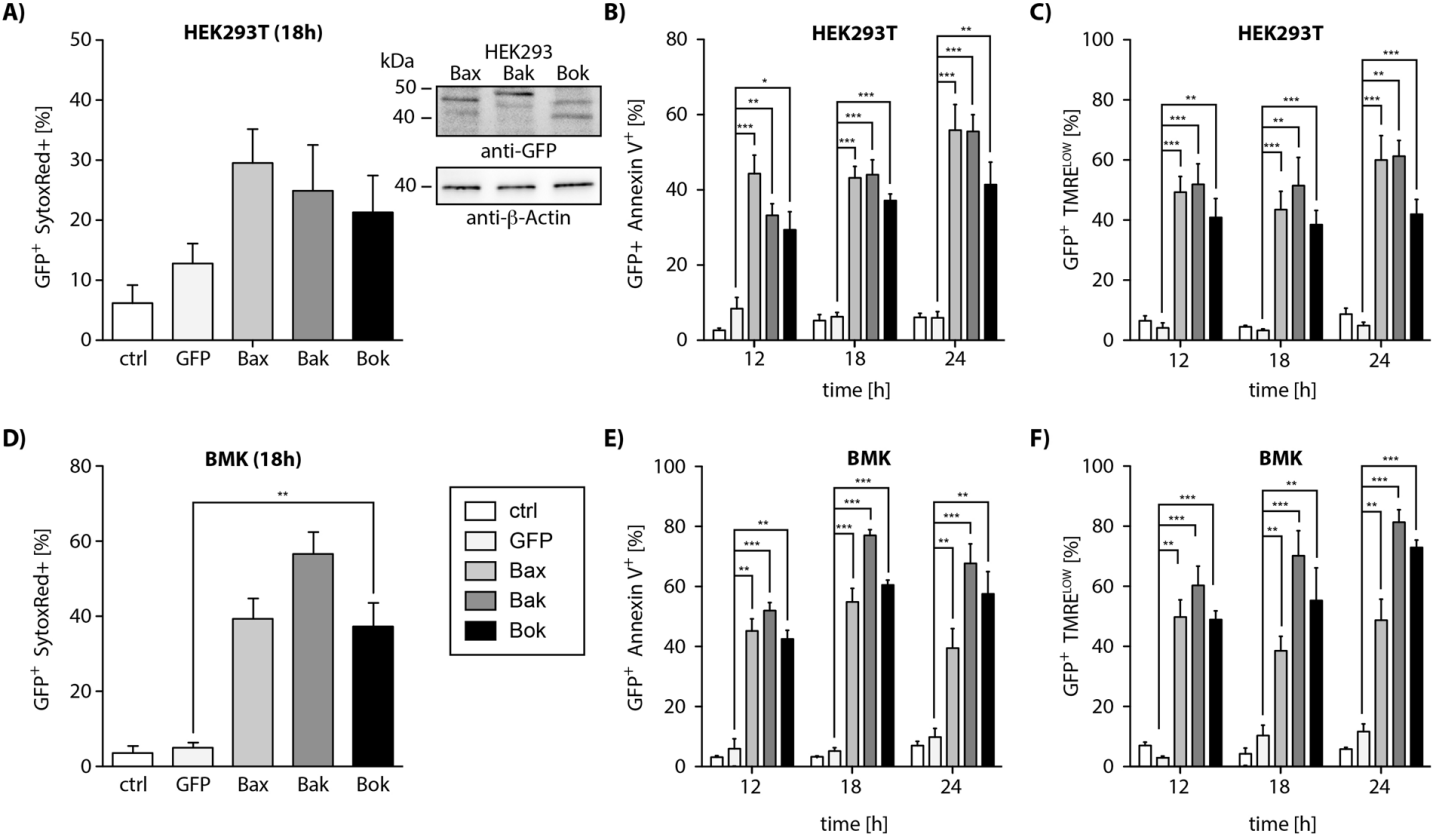
Flow cytometric analysis of HEK293 (**A**–**C**) cells expressing EGFP-fusion variants of Bax, Bak and Bok (representative western blot inlay; identical membrane was incubated with anti-GFP and anti-β-actin antibody sequentially) show induction of cell death (SytoxRed positive cells). Expression of EGFP-Bax, -Bak and -Bok in HEK293T cells induces (**B**) exposure of phosphatidylserine (PS) detected by Annexin V^+^-binding and (**C**) loss of mitochondrial membrane potential (TMRE^low^) at different time points. (**D**) EGFP-Bax, -Bak or -Bok expression induces cell death of Bax&Bak deficient BMK cells. Cell death induction in Bax&Bak deficient BMK cells by EGFP-tagged Bax-, Bak and Bok is associated with (**E**) exposure of PS and (**F**) loss of mitochondrial membrane potential at different time points.

Discrepancies in published data regarding the pro-apoptotic activity of Bok and its regulation encouraged us to compare the effect of Bok overexpression to that of Bax and Bak. We transfected HEK293T cells with vectors for the expression of EGFP fusion proteins of Bax, Bak and Bok. We harvested HEK293T cells 18 h post transfection and detected cell death by flow cytometric analysis of SytoxRed stained cells (Fig. 1A). As expected, overexpression of each of the multidomain proteins efficiently induced cell death in HEK293T cells, i.e. Bax (30%), Bak (25%) and Bok (20%). Importantly, in HEK293T cells there was no significant difference in cell death induction by Bok as compared to Bak. To further prove that cells die via apoptosis we performed identical experiments and investigated apoptosis associated exposure of phosphatidylserine by labeling cells 12 h, 18 h and 24 h post transfection with Annexin-V-APC. Subsequent flow cytometric analysis showed that each of the multidomain proteins efficiently induced exposure of phosphatidylserine at different time points in 45–55% (Bax), 35–55% (Bak), and 30–40% (Bok) of transfected cells showing slight differences in kinetics (Fig. 1B). We additionally assessed loss of the mitochondrial membrane potential in transfected cells by TMRE staining. Overexpression of each of the MDPs induced depolarization of mitochondria; the proportion of cells with low mitochondrial membrane potential at the indicated time points was 50–60% (Bax), 50–70% (Bak), or 40% (Bok) (Fig. 1C).

Having shown that the EGFP-fusion proteins of Bax, Bak and Bok induce apoptosis to a comparable extent in Bax and Bak proficient HEK293T cells we next investigated whether the MDPs also induce apoptosis in baby mouse kidney cells (BMK) from Bax^−/−^/Bak^−/−^ double knock out (DKO) mice. In line with recently published data^6^ overexpression of EGFP-fusion proteins of Bax, Bak and also Bok efficiently induced cell death in these BMK/DKO cells (Bax: 40%/; Bak: 55%; Bok: 40%) (SytoxRed^+^, Fig. 1D). Expression of EGFP-fused MDPs also induced exposure of phosphatidylserine (Annexin-V^+^; Fig. 1E) and dissipation of the mitochondrial membrane potential (TMRE^LOW^; Fig. 1F) in BMK cells at the indicated time points.

**Figure 2:**
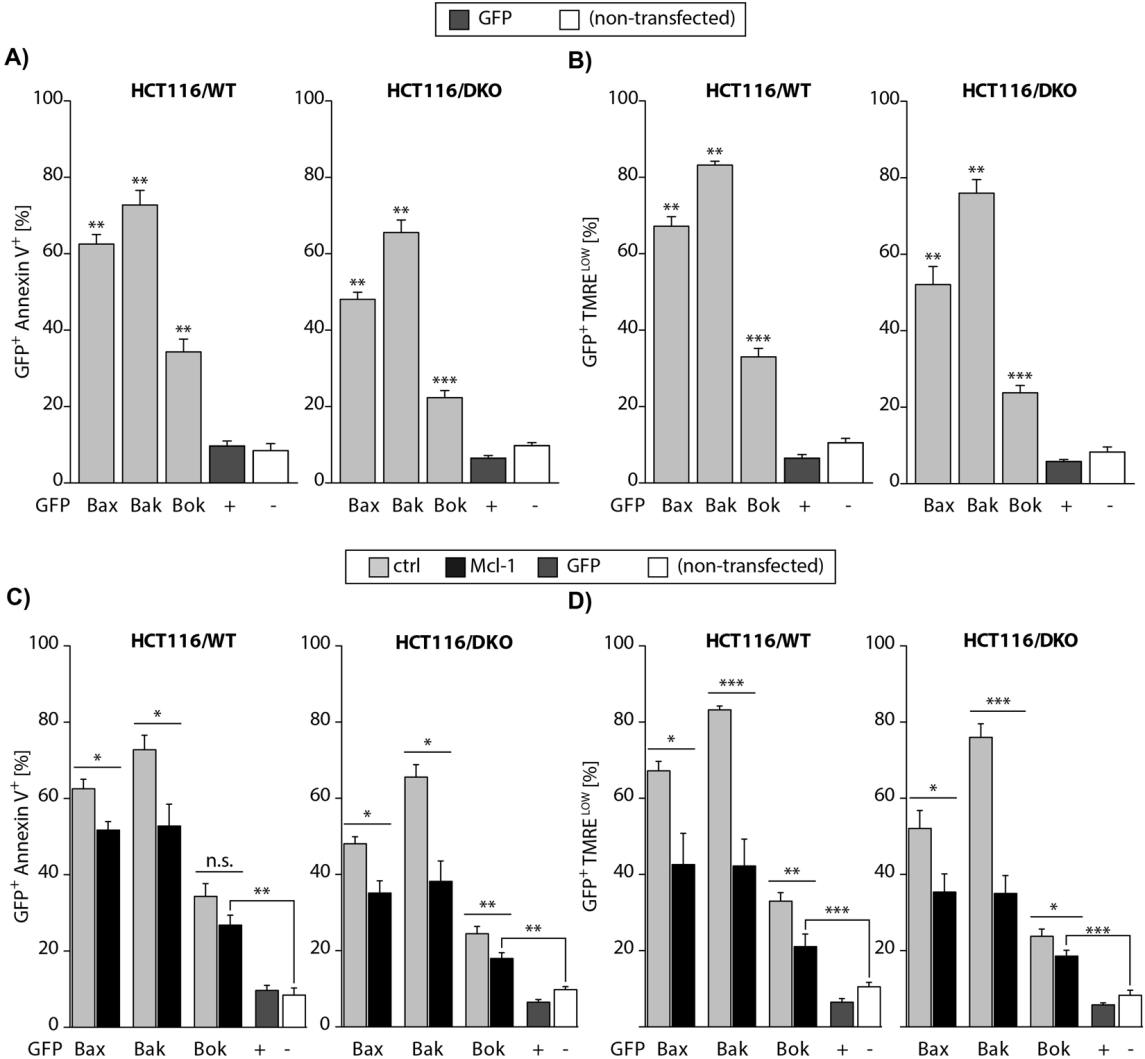
(**A**) Flow cytometric analysis of EGFP-Bax, -Bak or Bok expressing HCT116/WT and HCT116/DKO cells shows apoptosis associated exposure of phosphatidylserine by Annexin-V-APC staining. (**B**) Expression of EGFP-Bax, -Bak or –Bok is associated with decrease of the mitochondrial membrane potential in the presence (WT) and absence (DKO) of Bax&Bak shown by TMRE (TMRE^low^). (**C**) Number of Annexin-V-APC positive apoptotic cells is reduced by co-expression of mCherry-Mcl-1 as compared to EGFP-Bax, -Bak or –Bok expression alone in the presence (WT) and absence (DKO) of Bax and Bak. (**D**) Number of EGFP-Bax, -Bak or -Bok expressing cells showing dissipation of the mitochondrial membrane potential (TMRE^low^) is reduced by co-expression of mCherry-Mcl-1 independent of Bax&Bak.

Hence, in BMK cells Bok induces apoptosis independently of Bax and Bak whereas it was proposed to depend on the presence of Bax or Bak in HCT116 cells^14^. To prove independent apoptosis induction by Bok we repeated the experiments in Bax&Bak proficient (WT) and deficient (DKO) HCT116 cells. We found that each multidomain protein readily induced apoptosis in both cell lines, HCT116/WT and HCT116/DKO (Fig. 2A). Clearly, Bok does not rely on another pro-apoptotic MDP to induce apoptosis. However, apoptosis induction was reduced in HCT116/DKO as compared to HCT116/WT, indicating that endogenously expressed Bax and Bak reinforce apoptosis induction by MDP overexpression. We further corroborated these data by analyzing loss of mitochondrial membrane potential in transfected HCT116/WT and HCT116/DKO cells. To this end TMRE stained cells were analyzed by flow cytometry. The results show that overexpression of each MDP induced dissipation of the mitochondrial membrane potential in both cell lines (Fig. 2B). Exposure of phosphatidylserine (PS) and dissipation of the mitochondrial membrane potential was more efficiently induced in the presence of endogenous Bax and Bak (HCT116/WT) than in the Bax&Bak deficient HCT116/DKO. These results unambiguously establish that Bok induces loss of mitochondrial membrane potential and apoptosis in the absence of another pro-apoptotic multidomain protein. The discrepancy to published data, i.e. non-autonomous apoptosis induction by Bok in MEF/DKO, most likely is due to the expression level of Bok in combination with the expression level of anti-apoptotic (Bcl-2 like) proteins, such as Mcl-1.

### Mcl-1 counteracts apoptosis induction by Bax, Bak, and Bok

Indeed, interaction of Bok with Mcl-1 was proposed in the original publication that identified Bok as a pro-apoptotic Bcl-2 Protein^11^. Later, Echeverry *et al*. found reduced survival of Bok expressing Mcl-1^−/−^ mouse embryonic fibroblasts (MEFs) as compared to WT MEFs but did not detect interaction of HA-tagged Bok with Flag-tagged Mcl-1 in immunoprecipitation experiments^14^. Recently, Llambi *et al*. found that Mcl-1 delayed Bok mediated LUV permeabilization and co-precipitated with Bok but Mcl-1 did not affect Bok mediated cell death induction by MG132-induced proteasome inhibition in HCT116/DKO cells. Interestingly, in these studies Bok co-precipitated with Mcl-1 although the binding constant of the Bok-BH3 peptide to Mcl-1 was rather weak (3.0 µM)^15^. In order to clarify whether or not Mcl-1 counteracts Bok’s pro-apoptotic activity we co-transfected HEK293T cells with vectors for the expression of EGFP-fused Bax, Bak or Bok together with expression constructs for Flag-tagged Mcl-1 and then stained cells with TMRE and Annexin-V-APC for flow cytometric analysis. Mcl-1 significantly reduced induction of cell death (Fig. S1A left panel) and loss of mitochondrial membrane potential (Fig. S1A right panel) induced by its interaction partner Bak, and attenuated the function of Bax and Bok. Hence, we assumed the involvement of downstream activation of Bak in HEK293T. In order to discriminate interaction of overexpressed and endogenous MDPs with transgenic Mcl-1 we changed the experimental system and transfected HCT116/WT as control and HCT116/DKO cells with expression vectors for EGFP-tagged MDP proteins Bax, Bak and Bok together with expression constructs for N-terminally Flag-tagged Mcl-1.

MDP expression induced exposure of Annexin-V and loss of mitochondrial membrane potential in HCT116/WT and, HCT116/DKO cells (Fig. 2B). Co-expression of Mcl-1 reduced apoptosis induction by Bak and to a lesser extent that mediated by Bax and Bok (Fig. 2C). In HCT116/DKO cells the number of Annexin-V positive cells was significantly reduced by Mcl-1 co-expression in case of each MDP, Bax, Bak and also Bok (Fig. 2C, right panel). Furthermore, dissipation of the mitochondrial membrane potential (Fig. 2D) induced by each MDP was reduced by Mcl-1 in the presence (Fig. 2D, left panel) and absence of endogenous Bax&Bak (Fig. 2D, right panel). Expectedly, Mcl-1 counteracts Bak-mediated apoptosis while the inhibitory effect of Mcl-1 on Bok is more pronounced in HCT116/DKO cells due to the absence of downstream amplification by Bak.

### Impact of TM-deletion on apoptosis induction by MDPs

**Figure 3:**
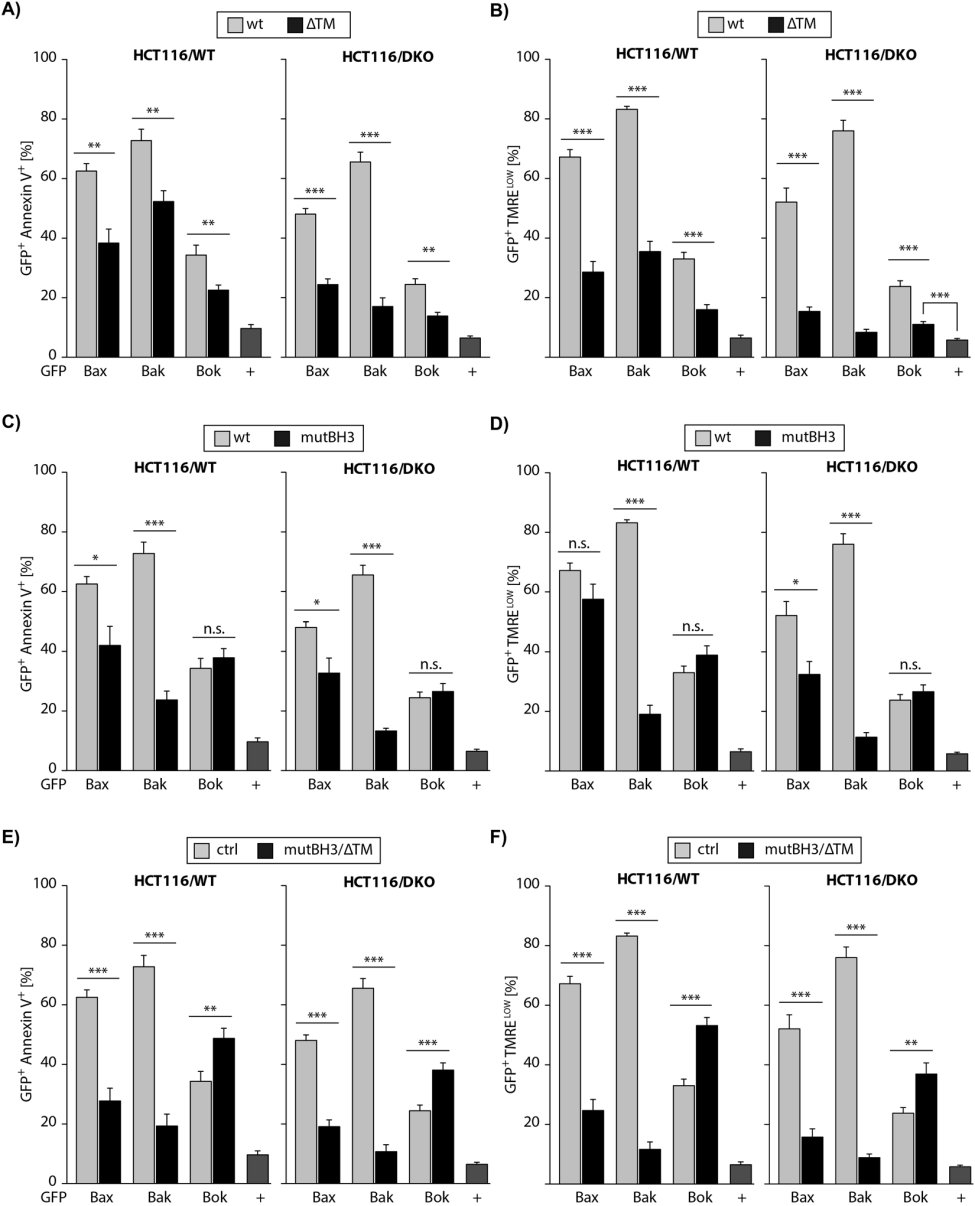
Deletion of the transmembrane-domain of Bax, Bak or Bok reduces the number of (**A**) apoptotic (Annexin-V-APC positive) and (**B**) cells with lost ΔΨm (TMRE^low^) in the presence (WT) and absence (DKO) of Bax&Bak. (**C**) Mutation of the conserved lysine residue in the BH3-domain reduces Bax-induced apoptosis, abolishes Bak-induced apoptosis but has no significant impact on Bok-induced apoptosis in the presence and absence of endogenous Bax&Bak. (**D**) The number of cells with lost mitochondrial membrane potential is slightly reduced by Bax(L63E), heavily reduced by BakL78E but unchanged by Bok(L70E) both, in HCT116/WT and HCT116/DKO. (**E**) Combined mutation of the BH3-domain and deletion of the transmembrane-domain severely dampens apoptosis induction by Bax(L63E)ΔTM and Bak(L78E)ΔTM but enhances cell death induction by Bok(L70E)ΔTM. (**F**) Bax(L63E)ΔTM and Bak(L78E)ΔTM are almost incapable to induce loss of ΔΨm whereas the number of TMRE^low^ cells is increased in case of Bok(L70E)ΔTM irrespective of the presence (WT) or absence (DKO) of Bax and Bak.

Having established inhibition of Bok-induced apoptosis by Mcl-1 we next investigated the molecular basis for Bok mediated apoptosis with regard to structural premises. We transfected cells with a vector for the expression of Bok variant that is deficient in the C-terminal transmembrane-domain (ΔTM). Again, we included BaxΔTM and BakΔTM in order to conclude on similarities or differences in the mechanism of apoptotsis induction. Transfection of HCT116/WT and HCT116/DKO and flow cytometric analysis of EGFP^+^ cells revealed, compared to the respective full length (FL) protein, a reduced proportions of Annexin-V^+^ HCT116/WT cells upon expression of BaxΔTM (60% vs. 40%), BakΔTM (70% vs. 50%) and BokΔTM (30% vs. 20%) (Fig. 3A, left panel). In HCT116/DKO the decrease of apoptotic cells due to deletion of the transmembrane-domain was more pronounced: BaxΔTM (50% vs. 25%), BakΔTM (65% vs. 20%) and BokΔTM (20% vs. 10%) (Fig. 3A, right panel). Accordingly, dissipation of the mitochondrial membrane potential by BaxΔTM, BakΔTM or BokΔTM was significantly reduced as compared to FL proteins both, in HCT116/WT and most distinctive in HCT116/DKO (Fig. 3B). We conclude that the transmembrane-domain has a significant impact on the pro-apoptotic potential of each multidomain protein, most prominently for the membrane localized Bak and slightly less pronounced in case of Bax and Bok.

### Impact of BH3 mutation on apoptosis induction by MDPs

The pro-apoptotic activity of MDP proteins also depends on their BH3-domain^19–21^. The α-helical BH3-domain (α2-helix) is highly conserved among Bcl-2 proteins and is characterized by hydrophobic residues that face in the same direction^22^ and thereby can mediate binding to the hydrophobic groove of Bcl-2 proteins^23^. Mutation of (the first) conserved hydrophobic amino acid in Bak^19^ and Bax^20^ results in decreased apoptosis induction because the BH3-domain is involved in homo-oligomerization^21^. Indeed, highly sophisticated mutational analyses revealed that the BH3-domain mediates Bax dimerization^24^. To analyze the role of the BH3-domain in Bok-induced apoptosis we transfected expression vectors for BH3-mutant Bax(L63E), Bak(L78E) and Bok(L70E) in HCT116/WT and HCT116/DKO cells and analyzed apoptosis induction (Annexin-V binding) and loss of ΔΨm (decreased TMRE fluorescence) by flow cytometry. Expectedly, we found reduced apoptosis induction by Bax(L63E) (62% to 42% in WT; 48% to 33% in DKO) and almost abolished cell death induction by Bak(L78E) (73% to 23% in WT; 66% to 13% in DKO; Fig. 3C). In contrast, apoptosis induction by Bok(L70E) was not reduced compared to Bok (34% to 38% in WT; 25% to 27% in DKO; Fig. 3C). Similarly, overexpression of Bax(L63E) showed attenuated dissipation of the mitochondrial membrane potential (67% to 57% in WT; 52% to 32% in DKO) and Bak(L78E) almost completely failed to induce loss of ΔΨm (83% to 19% in WT; 76% to 11% in DKO), compared to the respective WT proteins. Surprisingly and in contrast to Bax and Bak, Bok(L70E) induced loss of ΔΨm as efficiently as wildtype Bok (33% to 39% in WT; 24% to 27% in DKO) (Fig. 3D).

### Enhanced apoptosis induction by Bok(L70E)ΔTM

Deletion of the TM reduced apoptosis induction by each multidomain protein, including Bok, whereas BH3 mutation reduced Bax- and abolished Bak-induced apoptosis but did not affect activity of Bok. We next asked whether the combined deletion of TM and mutation of BH3 would further reduce the activity of EGFP-tagged Bax, Bak or Bok. Upon expression of the respective proteins, FACS analysis showed that Bax-induced apoptosis is strongly reduced by the L63E mutation in combination with the deletion of the TM (Fig. 3E). Bax(L78E)ΔTM induced apoptosis also is reduced compared to BaxΔTM or Bax(L63E), respectively (Fig. 3A,C,D). Because Bak(L78E) (Fig. 3C,D) was almost inactive no further reduction of apoptosis induced by Bak(L78E)ΔTM was detected in Bax/Bak proficient or deficient cells (Fig. 3E). Intriguingly, expression of Bok(L70E)ΔTM, compared to expression of Bok, Bok(L70E) and BokΔTM elicited significantly higher numbers of apoptotic cells in both, HCT116/WT (34% vs. 49%) and HCT116/DKO (25% vs. 38%) (Fig. 3E). The inactivation of Bax(L63E)ΔTM and Bak(L78E)ΔTM was also readily evident in FACS analyses of TMRE stained HCT116 cells (Fig. 3F) showing that both proteins fail to induce MOMP. Again, in contrast to Bax(L63E)ΔTM and Bak(L78E)ΔTM, also the loss of ΔΨm induced by Bok(L70E)ΔTM was enhanced as compared to WT Bok in HCT116/WT (33% vs. 53%) and HCT116/DKO (24% vs. 37%).

### Mcl-1 inhibition of MDP derivatives

**Figure 4:**
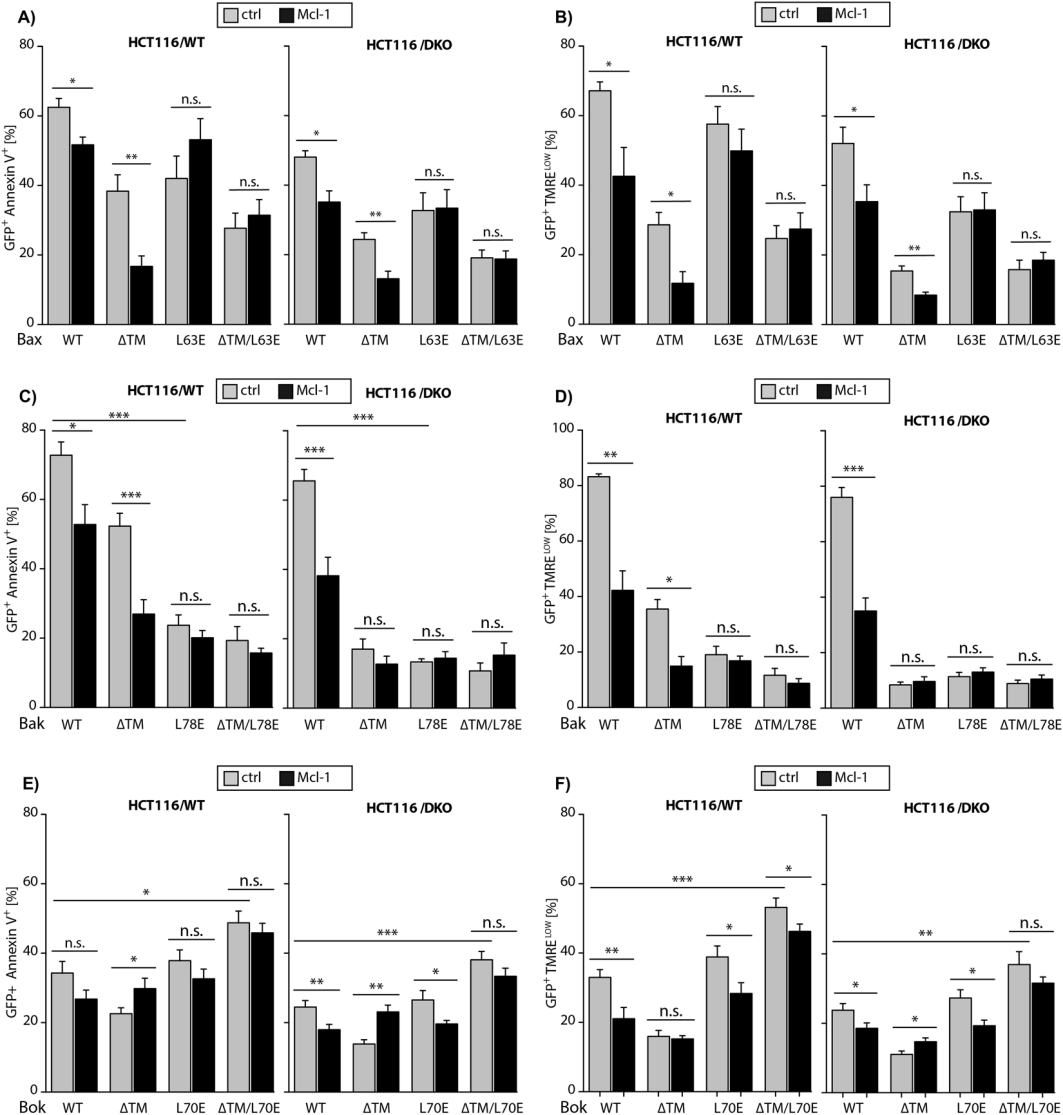
(**A**) Co-expression of Mcl-1 reduces apoptosis induction by Bax and BaxΔTM but has no impact on Bax(L63E) and Bax(L63E)ΔTM induced apoptosis in HCT116/WT and HCT116/DKO cells. (**B**) Loss of ΔΨm due to expression of Bax and BaxΔTM is reduced by co-expression of Mcl-1 but unchanged in case of Bax(L63E) and Bax(L63E)ΔTM. (**C**) Bak induced apoptosis is reduced by co-expression of Mcl-1 in HCT116/WT and HCT116/DKO whereas BakΔTM induced apoptosis is only reduced by Mcl-1 in HCT116/WT but not in HCT116/DKO. Bak(L78E) and Bak(L78E)ΔTM only marginally induce apoptosis that is not influenced by Mcl-1 co-expression. (**D**) Bak and BakΔTM induced dissipation of the mitochondrial membrane potential is reduced by Mcl-1 in HCT116/WT. In HCT116/DKO Bak induced loss of the mitochondrial membrane potential is reduced by Mcl-1 expression. (**E**) In HCT116/WT cells Mcl-1 expression has no significant impact on apoptosis induction by Bok, BokΔTM, Bok(L70E) and Bok(L70E)ΔTM. In the absence of endogenous Bax&Bak co-expression of Mcl-1 reduces apoptosis induction by Bok and Bok(L70E) whereas BokΔTM and Bok(L70E)ΔTM induced apoptosis is not influenced by Mcl-1 in HCT116/DKO. (**F**) Dissipation of the mitochondrial membrane potential in Bok, Bok(L70E) and Bok(L70E)ΔTM expressing HCT116/WT cells is reduced by Mcl-1 co-expression. In the absence of Bax&Bak dissipation of the mitochondrial membrane potential is reduced by Mcl-1 in Bok and Bok(L70E) expressing cells whereas BokΔTM and Bok(L70E)ΔTM induced loss of the mitochondrial membrane potential is not affected by Mcl-1 expression.

Puzzled by the unexpected enhanced activity of Bok(L70E)ΔTM we wondered whether this might be caused by reduced inhibition by (endogenously expressed) anti-apoptotic Bcl-2 proteins, e.g. Mcl-1. We therefore analyzed HCT116/WT and DKO cells co-expressing the mutant variants of Bax, Bak, or Bok together with Flag-Mcl-1. Flow cytometric analysis showed that Mcl-1 slightly attenuates apoptosis induction by Bax and more pronounced apoptosis induction by BaxΔTM indicated by a reduced number of Annexin-V^+^ and ΔΨm^LOW^ cells (Fig. 4A,B). Expression of Bax(L63E) and Bax(L63E)ΔTM resulted in similar numbers of Annexin-V^+^ and ΔΨm^LOW^ cells irrespective of co-expressed Mcl-1. Thus, mutation of Bax BH3 abolishes the (weak) interaction of Bax with Mcl-1. The anti-apoptotic effect of Mcl-1 co-expression was most pronounced upon overexpression of Bak (Fig. 4C). Mcl-1 also reduced apoptosis induction by BakΔTM in HCT116/WT cells (Fig. 4C,D, left panels), however, most likely due to downstream inhibition of endogenous Bak (and/or Bax), because neither expression of BakΔTM, Bak(L78E) or Bak(L78E)ΔTM did induce apoptosis in HCT116/DKO cells (Fig. 3). Consequently, in HCT116/DKO cells there was no measurable impact of Mcl-1 co-expression on BakΔTM- or Bak(L78E)-induced apoptosis (Fig. 4C,D) and no conclusions can be drawn with regard to the inhibition of BakΔTM, Bak(L78E) or Bak(L78E)ΔTM by Mcl-1. Finally, WT Bok and also Bok(L70E)-induced apoptosis was blocked by Mcl-1 (Fig. 4E,F) whereas Mcl-1 did not reduce apoptosis induced by BokΔTM or Bok(L70E)ΔTM. We conclude that Bok(L70E) still interacts with Mcl-1 whereas deletion of the TM-domain abolishes interaction of Bok and Mcl-1. The enhanced apoptosis induction by Bok(L70E)ΔTM as compared to Bok in HCT116/DKO might reflect auto-inhibition of Bok by the TM-domain potentially folded back onto inactive Bok or reduced inhibition by endogenous Mcl-1 (or other anti-apoptotic Bcl-2 proteins) because inhibition of Bok is no longer effective upon TM deletion (Fig. 4E,F, right panels). The anti-apoptotic effect of Mcl-1 on Bok(L70E)ΔTM in HCT116/WT most likely reflects downstream inhibition of Bak (Fig. 4E,F, left panels).

### Determinants of Bok and Mcl-1 interaction

**Figure 5:**
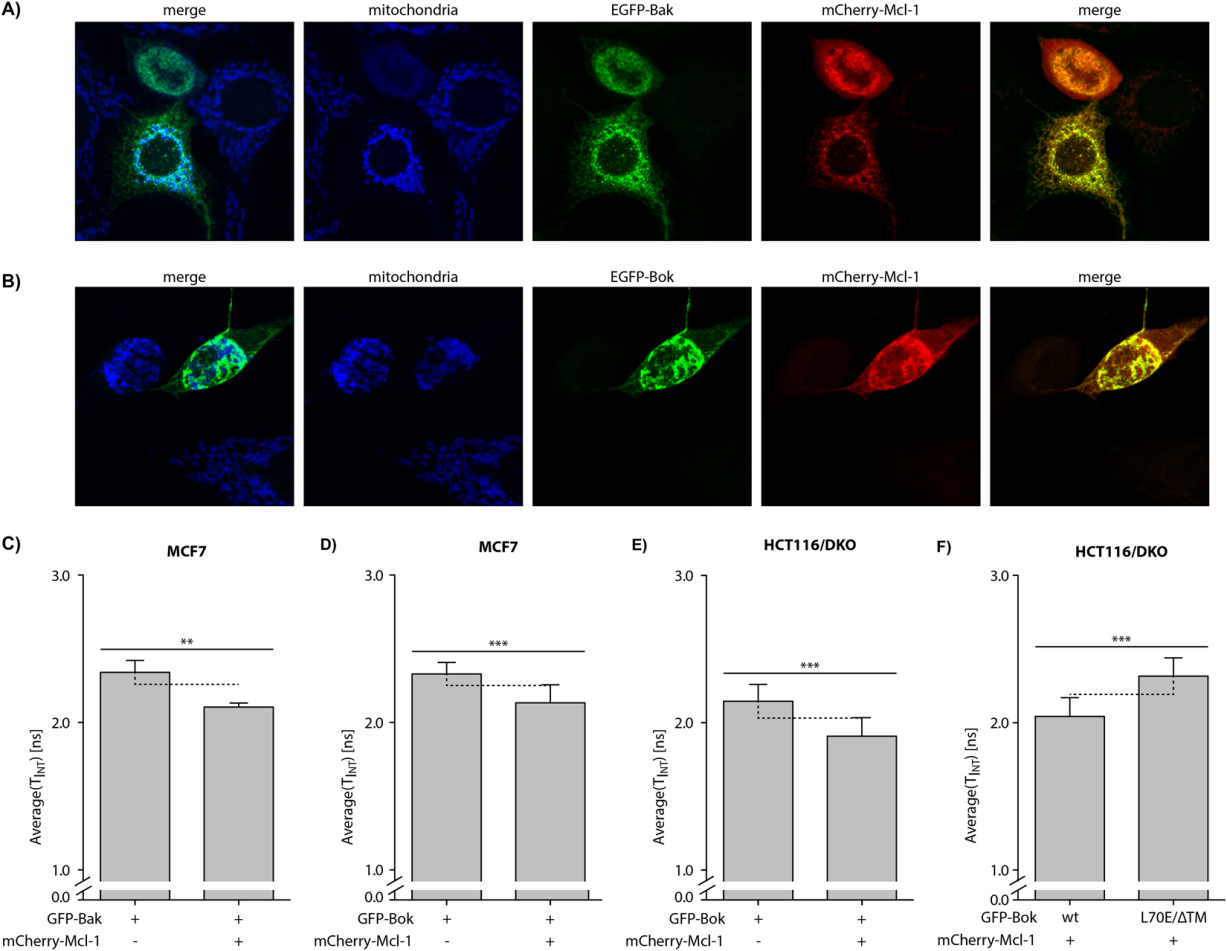
Confocal laser scanning microscopy of cells co-expressing (**A**) EGFP-Bak or (**B**) EGFP-Bok together with mCherry-Mcl-1 reveals EGFP-cluster formation, co-localization of EGFP-Bak or EGFP-Bok with mCherry-Mcl-1 (merge) and the mitochondria (Tom20, blue). (**C**–**F**) FRET/FLIM analysis shows reduction of fluorescence lifetime by mCherry-Mcl-1 in MCF7 cells for (**C**) EGFP-Bak and (**D**) EGFP-Bok. In HCT116/DKO cells co-expression of mCherry-Mcl-1 results in reduced fluorescence lifetime of EGFP-Bok. *Vice versa*, EGFP-Bok(L70E)ΔTM has a longer fluorescence lifetime than EGFP-Bok in mCherry-Mcl-1 co-expressing HCT116/DKO cells. Error bars represent average lifetime ±SD of n > 10 cells analyzed.

To investigate the localization of Bok and the interaction of Bok with Mcl-1 we performed immunofluorescence microscopy in MCF7 cells because of their inherent apoptosis resistance downstream of MOMP due to caspase-3 deficiency^25^. In control experiments overexpression of EGFP-Bak together with mCherry-Mcl-1 readily showed localization of EGFP-Bak at mitochondria and co-localization of EGFP-Bak with mCherry-Mcl-1 (Fig. 5A). Similarly, EGFP-Bok localized to mitochondria and co-localized with mCherry-Mcl-1 (Fig. 5B). Because co-localization is a prerequisite for protein-protein interaction but does not prove binding of Bok to Mcl-1 we performed fluorescence/Förster resonance energy transfer (FRET) fluorescence lifetime imaging (FLIM) analysis. In FRET-FLIM analysis the fluorescence lifetime of the FRET-donor (EGFP) is reduced in case a FRET-acceptor (mCherry) is in very close proximity. We transfected MCF7 cells with vectors for the expression of EGFP-Bak or EGFP-Bok alone (as control) and in combination with mCherry-Mcl-1. FRET/FLIM analysis revealed a fluorescence lifetime of τ = 2.36 ± 0.04 ns for EGFP-Bak that was shortened to τ = 2.11 ± 0.03 ns in the presence of its mCherry-tagged binding partner Mcl-1 (Fig. 5C). The fluorescence lifetime of EGFP-Bok was τ = 2.34 ± 0.06 ns and comparably reduced by 0.20 ns in the presence of mCherry-Mcl-1 (τ = 2.14 ± 0.12 ns, Fig. 5D). These results strongly indicate direct interaction of Bok with Mcl-1. Confirming these data, also FRET/FLIM analysis in HCT116/DKO showed that the fluorescence lifetime of EGFP-Bok was reduced from τ = 2.14 ± 0.11 ns in the absence to τ = 1.91 ± 0.12 ns in the presence of mCherry-Mcl-1 (Fig. 5E). Hence, also in a Bax&Bak deficient system a direct interaction of Bok and Mcl-1 is evident. In view of the inability of Mcl-1 to attenuate apoptosis induction by Bok(L70E)ΔTM we analyzed the fluorescence lifetime of Bok and Bok(L70E)ΔTM in the presence of mCherry-Mcl-1. In HCT116/DKO the average fluorescence lifetime of EGFP-Bok in the presence of mCherry-Mcl-1 was τ = 2.04 ± 0.13 ns as compared to τ = 2.32 ± 0.12 ns for EGFP-Bok(L70E)ΔTM – clearly reflecting the reduced interaction of Bok(L70E)ΔTM with Mcl-1 (Fig. 5F). We furthermore measured also in Bax&Bak deficient BMK cells co-transfected with mCherry-Mcl-1 a shorter lifetime of τ = 2.19 ± 0.12 ns for EGFP-Bok than for EGFP-Bok(L70E)ΔTM (τ = 2.39 ± 0.26 ns; Fig. S2A). Finally, we tested whether the interaction of Bok and Mcl-1 also depends on the TM-domain of Mcl-1. To this end we analyzed MCF7 cells expressing EGFP-Bok alone, EGFP-Bok or EGFP-Bok(L70E)ΔTM together with mCherry-Mcl-1, or EGFP-Bok together with mCherry-Mcl-1ΔTM. We detected reduced EGFP fluorescence lifetime exclusively when TM-proficient Bok and TM-proficient Mcl-1 were co-expressed whereas the co-expression of EGFP-Bok(L70E)ΔTM and mCherry-Mcl-1 and importantly also EGFP-Bok and mCherry-Mcl-1ΔTM did not result in reduced fluorescence lifetime of EGFP (Fig. S2B). Hence, we propose that the efficient inhibition of Bok induced apoptosis by Mcl-1 depends on the presence of the transmembrane-domain in both proteins, i.e. Bok and Mcl-1.

In summary we show that Bok induces cytochrome c release and apoptosis independent of Bax and Bak. Furthermore, apoptosis induction by Bok is reduced by overexpression of Mcl-1. The most likely mechanism for inhibition of Bok-induced apoptosis by Mcl-1 is direct interaction via the TM-domain, because deletion of the TM-domain abolishes inhibition of Bok-induced apoptosis by Mcl-1. This is corroborated by FRET-FLIM analyses that show reduced fluorescence lifetime of EGFP-Bok in the presence of mCherry-Mcl-1. *Vice versa*, in the presence of Mcl-1 the fluorescence lifetime of EGFP-Bok(L70E)ΔTM is increased compared to EGFP-Bok indicating abolished interaction. Hence, Bok is a genuine pro-apoptotic multidomain Bcl-2 protein that mediates release of cytochrome c from the mitochondria and that is counteracted by the anti-apoptotic protein Mcl-1. We furthermore show that for efficient interaction of Bok with Mcl-1 the transmembrane-domains of both proteins are essential.

## Discussion

In the past 30 years highly sophisticated analyses have disclosed numerous details of how members of the Bcl-2 family regulate apoptosis in a complex network of specific interactions. The downstream effectors of the Bcl-2 network, i.e. Bax and Bak, undergo a series of distinct conformational changes and finally permeabilize the mitochondrial outer membrane by forming oligomeric structures^3^. The Bax/Bak homologous protein Bok however, seems to withstand consistent characterization^26^ as seemingly conflicting results are published regarding its ability to autonomously induce apoptosis and the interaction of Bok with anti-apoptotic proteins, such as Mcl-1. Echeverry *et al*.^14^ published that Bok cannot induce apoptosis in the absence of Bax and Bak whereas Einsele-Scholz *et al*.^6^ convincingly showed Bok-induced apoptosis in the absence of Bax and Bak. The latter was recently confirmed by Llambi *et al*. who published that Bok induces apoptosis and MOMP in the absence of Bax or Bak albeit not inhibited by any of the anti-apoptotic Bcl-2 proteins. All three authors used different model systems, i.e. MEF (Echeverry) or BMK (baby mouse kidney cells, Einsele-Scholz) from Bax/Bak double knock out mice or HCT116/DKO cells^18^. Also, Bok was either inducibly expressed (Echeverry: 4-OHT, Llambi: Doxycycline) or controlled by a CMV-promotor. It is conceivable that the expression level achieved by inducible expression does not overcome the threshold of endogenously expressed anti-apoptotic proteins – especially in the absence of Bax and Bak. In this line, the potential criticism addressed at the non-physiological nature of overexpression is not applicable in case of overexpression in knock-out models, as KO-models do not display a physiological background themself. Self-evidently, Bok induces apoptosis more efficiently in the presence of Bax and Bak than in their absence – due to co-operative action or a reduced threshold of free anti-apoptotic proteins. However, the same is true for overexpressed Bax and to some extent also for Bak, as shown in Figs 1–4.

Here, we investigated whether the presence of anti-apoptotic proteins counteracts Bok-induced apoptosis and found that co-expression of the anti-apoptotic proteins Bcl-2 or Bcl-xL did not reduce Bok-induced apoptosis (not shown). In contrast, Mcl-1, that served as bait in the yeast two hybrid screen that identified Bok^11^, clearly reduced Bok-induced apoptosis. In order to dissect structural similarities and differences between Bax, Bak and Bok we analyzed induction of apoptosis upon expression of TM-deficient variants of these proteins. These experiments show that deletion of the TM-domain reduces the pro-apoptotic capacity of all three proteins. In this regard, Bok functions similar to Bax rather than Bak that shows reduced but not abolished pro-apoptotic activity upon removal of the TM-domain. Mutation of the conserved hydrophobic leucine in the BH3-domain reduced apoptosis induction by Bax and Bak, but unexpectedly had negligible impact on Bok-induced MOMP and apoptosis, indicating that Bok(L70E) still oligomerizes and forms pores. Despite these data underlining the structural and functional similarity of Bok to Bax and Bak, it is especially puzzling that simultaneous mutation of L70E and deletion of the transmembrane-domain enhanced rather than abolished apoptosis induction by Bok(L70E)ΔTM. The reason for this unexpected finding likely is that inhibition of Bok by Mcl-1 is not only mediated by the BH3-domain but also the TM-domain. This would mean that mutation of L70E reduces interaction of Bok with Mcl-1 and additional deletion of the TM-domain in Bok renders Bok unaffected by co-expression of Mcl-1. Then, Bok(L70E)ΔTM shows enhanced apoptosis induction as compared to Bok(L70E) because it is unaffected by endogenous Mcl-1.

Recently, Zheng *et al*. published the NMR derived structure of Bok (ΔN20-Bok-ΔC35) and proposed intrinsic structural instability of Bok. This instability spawns an active Bok conformation mediating mitochondrial membrane permeabilization by Bok^27^. In the proposed structure, the L70E mutation would further destabilize Bok(L70E)ΔTM and thereby augment occurrence of an active conformation. The resulting increased proportion of active Bok conformers fuels apoptosis detected as hyperactivity. Although the TM-domain is dispensable for apoptosis induction^15^ flow cytometric analyses show hyperactivity for Bok(L70E)ΔTM rather than Bok(L70E). Hence, the deletion of the TM-domain might additionally favor mitochondrial localization by shifting the balance from ER localized Bok to mitochondria where Bok mediates membrane permeabilization.

The proposed TM-domain-mediated interaction is further corroborated by FRET/FLIM data showing that Bok(L70E)ΔTM, in contrast to Bok, is not localized in close proximity to Mcl-1 (Fig. S2). A contribution of the TM-domain to the interaction with its anti-apoptotic binding partner was also shown for Bax and Bcl-2^28^. Interaction via the TM-domain would also explain co-precipitation with Mcl-1^15^ despite a rather low binding constant of the Bok-BH3 peptide for Mcl-1. Assuming structural similarity, the oligomerization of Bax-dimers by TM-domain interaction^24^ appears to be dispensable meaning that additional interaction *via* e.g. α1:α1, α3:α5 and α6:α6 would drive multimerization of BokΔTM (or BaxΔTM)^24,29,30^.

**Figure 6:**
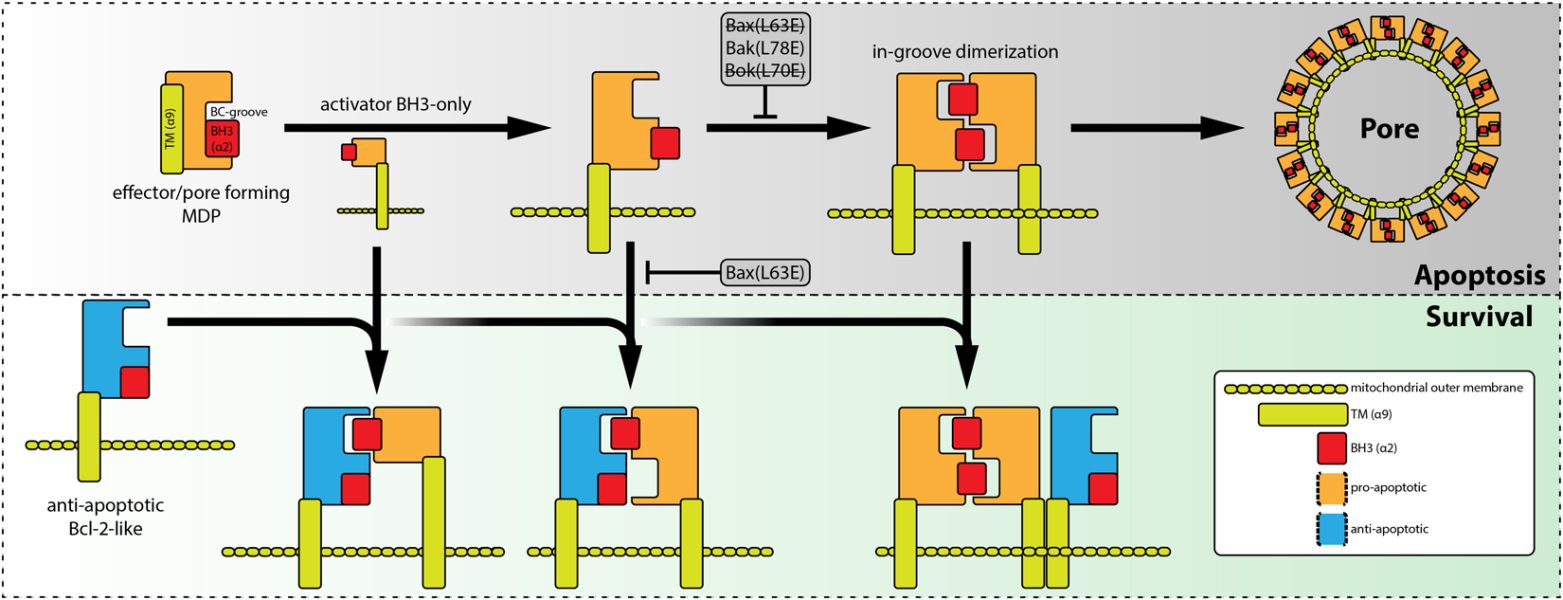
Schematic representation of the generalized model deduced from results in this study. Anti-apoptotic Bcl-2-like proteins interact with pro-apoptotic BH3-only or effector/pore-former proteins via BH3-domain/BC-groove binding and/or TM-domain – both resulting in inhibition of effector multimerization.

However, we propose that interaction of Bok with Mcl-1 is mediated both, by the TM-domain (α9) and the BH3-domain (α2). A schematic representation of the generalized model illustrates the interaction of effector/pore former MDPs and anti-apoptotic “Bcl-2” proteins via BH3/BC-groove binding and TM-domain interaction (Fig. 6).

Our data clearly show that Bok mediates apoptosis in the absence of Bax and Bak. The discrepancy in the conclusion whether Bok autonomously induces cytochrome c release^6^ or is only capable to do so in the presence of Bax or Bak^14^ most likely is due to different expression levels of Bok in the context of varying disposable anti-apoptotic Bcl-2 proteins, especially Mcl-1. It is noteworthy to mention that Mcl-1 is quickly degraded by the proteasome^16,17^ and the stabilization of Bok by proteasome inhibitors^15^ therefore might be an indirect effect due to binding to Mcl-1. Similarly, stabilization of Mcl-1 is seen when the BH3-only proteins Puma^31^ or Nbk/Bik are overexpressed^4^. In case of the investigated experimental system using overexpression of GFP-fusion proteins it is worth to mention that inhibition of the proteasome by MG132 only marginally influences the expression level of GFP-Bok variants (Fig. S3A) whereas inhibition of caspase-activity, i.e. apoptosis, by Q-VD-OPh results in massively enhanced expression levels, especially of apoptosis inducing GFP-fused Bok variants (Fig. S3B). Also, the expression level of endogenous Bok is only slightly enhanced by different concentrations of MG132 after 8 h and 24 h whereas Mcl-1 expression levels are strongly increased at lowest concentration (Fig. S3C). In line with proteasomal degradation of endogenous Mcl-1 also the expression level of GFP-Mcl-1 is enhanced by MG132 whereas GFP-Bok expression is only marginally affected (Fig. S3D). Finally, the reduced detection of apoptosis inducing-proteins as compared to inactive-variants (Fig. S4A,B) is commonly seen and likely reflects apoptosis-associated reduced expression and/or protein loss due to permeabilization of the plasma membrane in secondary necrosis.

Although there are still some things about Bok we just don’t get^32^ it is likely that the seemingly incongruent results arise from the lacking incorporation of additional parameters of the different experimental systems, e.g. expression level of investigated proteins, especially in view of the dynamic nature of protein interactions. Looking at the long winding path to the development of solid models for the regulation of Bax and Bak activity^33^ that finally take relevant biochemical parameters, i.e. binding constants, into account, we are confident that comprehensive and more sophisticated analyses also will reveal the true nature of Bok in apoptosis.

## Materials and Methods

### Cell Culture

Cells were sub-cultured 2 times a week in the appropriate cell culture medium, supplemented with 10% fetal calf serum, 100 U/ml penicillin and 0.1 mg/mL streptomycin: HCT116/WT and HCT116/DKO in McCoy’s 5A; HEK293, DU145 and Baby Mouse Kidney cells from Bax/Bak KO mice (BMK) in Dulbecco’s Modified Eagle Medium (4,5 g/L glucose); MCF7 in Rosswell Park Memorial Institute (RPMI1640) Medium. Cells were seeded 24 h prior to transfection in 6-well or 12-well plates.

### Transfection

Transfection with the indicated vectors was achieved using Fugene6 (MCF7 and DU145) or JetPEI (HCT116, BMK) according to the manufacturer’s (Roche or Promega, respectively) protocol. If not indicated otherwise cells were harvested 18 h post transfection and processed for flow cytometric analysis or western blot. Plasmids have been published previously^6^ and sequences have been verified repeatedly to code for the canonical isoform of human Bax (Q07812), Bak (Q16611), Bok (Q9UMX3), Mcl-1 (Q07820) or mutants thereof.

### Flow Cytometry

Supernatant was collected and cells were detached from the plastic surface using 0.05% trypsin/EDTA. Detached cells were resuspended in supernatant, pelleted at 300 x g, washed in DPBS and resuspended in RPMI1640 w/o phenol red supplemented with 2% FCS and 0.4 µM tetramethylrhodamine ethyl ester. Samples were incubated at 37 °C for 30 min., spun down at 300 × g, washed in DPBS and resuspended in Annexin-V binding buffer supplemented with Annexin-V-APC. After incubation at RT for 15 min. cells were analyzed for EGFP-expression and Annexin-V-APC or TMRE fluorescence by flow cytometry using a LSR II (Becton Dickinson) and FACS Diva software. At least 10,000 EGFP^+^ cells were analyzed in each experiment.

### Statistical analysis

FACS data presented in bar graphs are mean ± SEM of n ≥ 3 independent biological experiments. Please note that mean values were calculated from independent sets of experiments and identical values (e.g. control, WT proteins w/o co-transfection) are presented repeatedly in different diagrams and figures for better comparability. Asterisks indicate *p < 0.05; **p < 0.01; ***p < 0.001. FLIM data are mean ± SD of n ≥ 7.

### Western blot

Cells were harvested by scraping, lysed in lysis buffer (1% Triton X-100, 10 mM Tris-HCl pH 7.5, 150 mM NaCl, 2 mM MgCl2 and 5 mM EDTA, pH 8.0) and relative protein content was assayed using the BCA protein assay (Pierce). Equal amounts of protein (30 µg) were separated in an SDS-polyacrylamide gel and transferred to nitrocellulose membrane using semi-dry blotting. Membranes were blocked in PBS/0.05% Tween20 (PBS-T) containing 5% BSA and incubated with primary antibody (0.2 μg/mL) in PBS-T/5% BSA at 4 °C over night. After washing thrice membranes were incubated with species specific horseradish peroxidase coupled antibody (Jackson Laboratories) in PBS-T/5% BSA for 2 h with shaking, washed in PBS-T and specific bands were visualized by enhanced chemiluminescence. Pictures were taken using the Fusion FX System (PeqLab) until sufficient pixel saturation was achieved or using x-ray films.

### Chemicals and antibodies

All chemicals were purchased in *pro analysi* grade from Merck KGaA (Germany) or Carl Roth GmbH. Antibodies for western blot were rabbit anti-Bok (Abcam; EPR15331), mouse anti-GFP (BioLegend; Clone B34) and rabbit anti-Tom20 (Santa Cruz Biotechnology; FL-145), mouse anti-*β*-actin (Sigma; clone AC74), anti-Mcl-1 (Santa Cruz Biotechnology, H-260) anti-mouse-HRP and anti-rabbit-HRP (Jackson Laboratories).

### Immunofluorescence microscopy and FRET/FLIM analysis

Cells were seeded on coverslips or in chamberslides 24 h prior to transfection. Immediately before transfection with the indicated plasmids for the expression of EGFP- and/or mCherry-fusion proteins culture medium was replaced by fresh medium supplemented with 10 µM Q-VD-OPh (MP Biomedicals). After 18 h or 20 h transfected cells were fixed in PBS/4% H_2_CO for 20 min. at room temperature and washed in cold PBS. For immunofluorescence microscopy fixed cells were incubated in specific antibody diluted in PBS/0.05% Saponin/5% BSA over night at 4 °C. Samples were washed in PBS two times and then incubated with species specific secondary antibody coupled to Alexafluor-405 or Alexafluor-633. Finally, samples were washed in PBS and mounted on coverslides.

For FRET/FLIM analysis coverslips were equilibrated in Na-phosphate buffer pH 8.5 and mounted using mounting medium (DAKO). Cells in chamberslides were stored in PBS/Tris (pH 8.5). The measurements were performed using a TCS SP8 confocal laser scanning microscope (Leica Microsystems) with LAS X and SymPhoTime software. Before the FRET-FLIM measurement, the presence of the fluorophores was detected using 488 and 561 nm lasers for GFP and mCherry excitation, respectively. The fluorescence lifetime (FLT) [ns] of either the donor only expressing cells or the donor-acceptor pairs was measured by excitation with a 470 nm pulsed laser at a repetition rate of 40 MHz (PicoQuant Sepia multichannel picosecond diode laser, PicoQuant Picoharp 300 TCSPC module, and Picosecond event timer). For each sample 500 photons in the brightest pixel of at least 7 cells each were acquired. Data processing was performed with SymPhoTime software for curve fitting, correction for the instrument response function, and a limited fitting range from channel 0 to 20 ns to obtain the GFP fluorescence lifetime.

## Electronic supplementary material

Supplementary Figures S1-4

## Data Availability

No datasets were generated or analyzed during the current study.
