# Supplementary material for: Contribution of BH3-domain and Transmembrane-domain to the Activity and Interaction of the Pore-forming Bcl-2 Proteins Bok, Bak, and Bax
Source: Sci Rep. 2018 Aug 20;8:12434. doi: 10.1038/s41598-018-30603-6 (PMC6102298; doi:10.1038/s41598-018-30603-6)
Supplement: Supplementary file 1 — Supplementary Figures S1-4 [file 41598_2018_30603_MOESM1_ESM.pdf]

Contribution of BH3-domain and Transmembrane-domain to the Activity and Interaction of the Pore-forming Bcl-2 Proteins Bok, Bak, and Bax

Daniel Stehle[1], Melanie Grimm[1], Stephanie Einsele-Scholz[1], Friederike Ladwig[2], Janina Johänning[3], Gerd Fischer[1], Bernhard Gillissen[4], Klaus Schulze-Osthoff[1,5], and Frank Essmann[1,*].

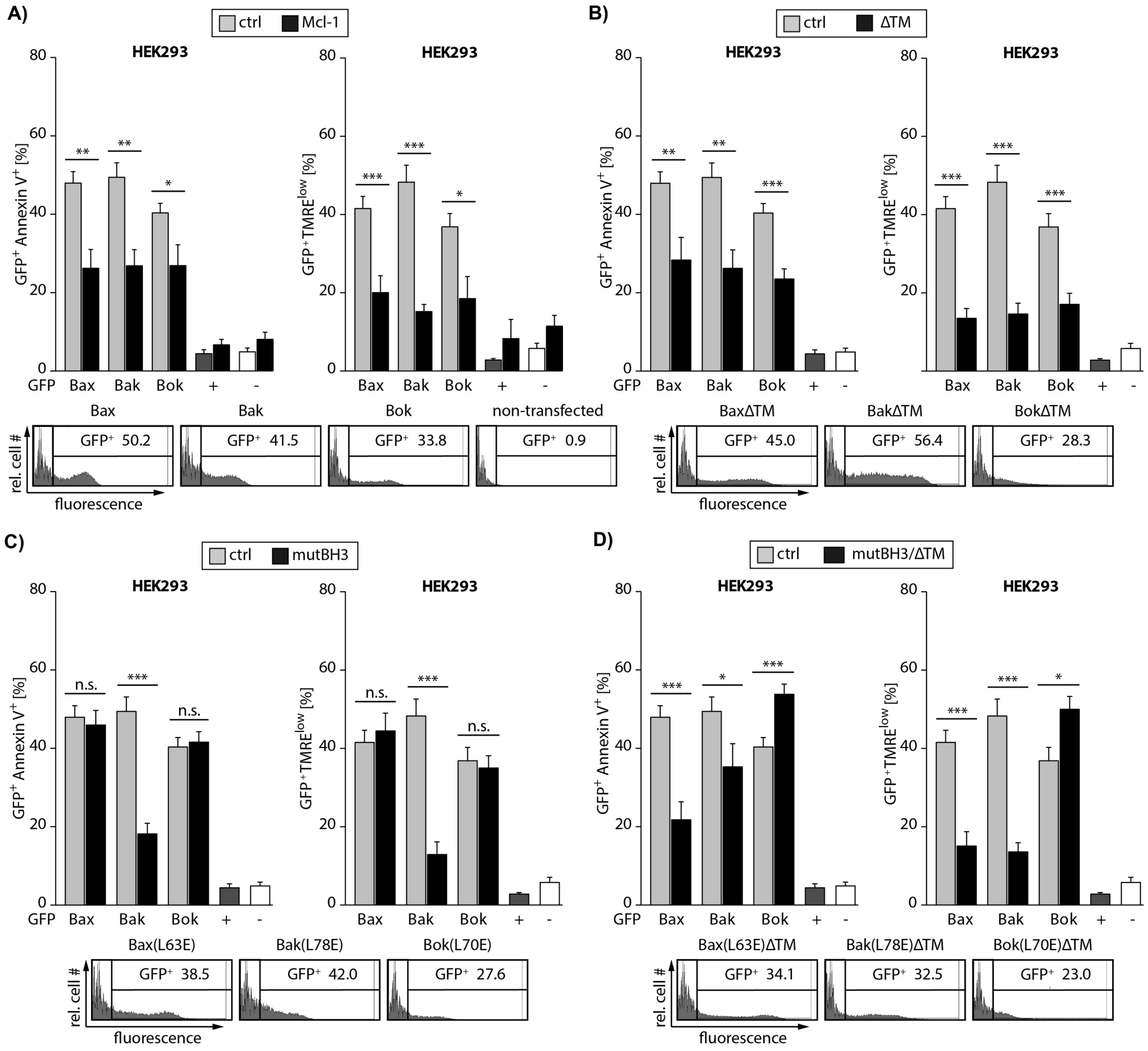

Supplemetal Figure S1: In HEK293 cells apoptosis induction (Annexin-V^+, left panels) and loss of $\Delta\Psi m$ ($TMRE^{low}$, right panels) by GFP-Bax, -Bak and -Bok is reduced by A) co-expression of Mcl-1, and B) deletion of the transmembrane-domain (ΔTM). C) Mutation of the BH3-domain exclusively diminishes cell death and loss of $\Delta\Psi m$ induced by GFP-Bak(L78E) but has no impact on GFP-Bax(L63E) and GFP-Bok(L70E). D) Simultaneous deletion of the TM-domain and mutation of the BH3-domain results in a reduced proportion of apoptotic cells (left panel) and cells with low TMRE-staining in case of Bax(L63E)ΔTM and Bak(L78E)ΔTM whereas Bok(L70E)ΔTM induced apoptosis is enhanced. Representative histograms below bar graphs show relative cell number and relative GFP-fluorescence intensity 18h post transfection. The proportion of GFP^+ gated cells is given in %.

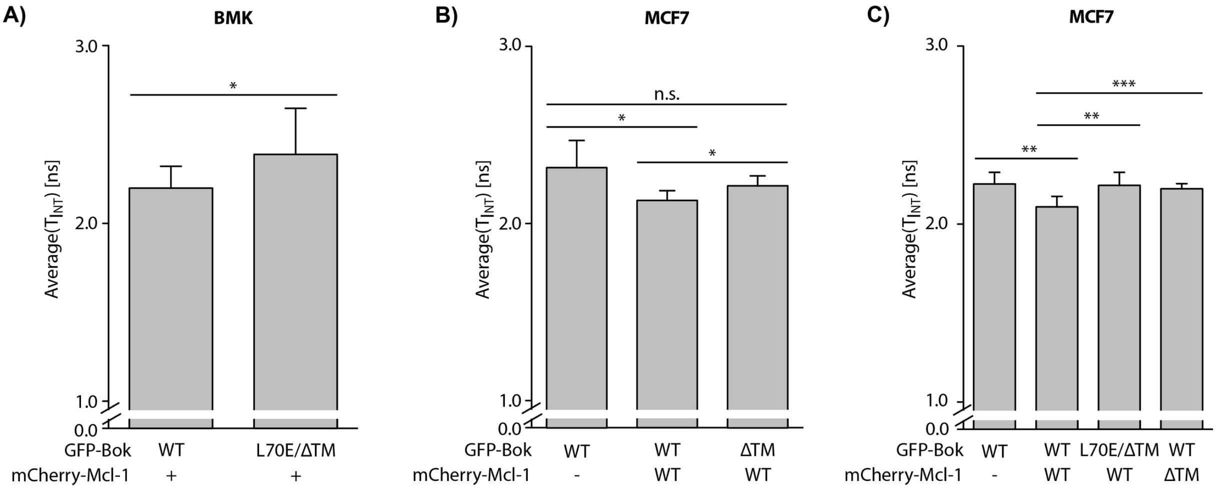

Supplemental Figure S2: A) FRET/FLIM analysis of Bax&Bak deficient BMK cells expressing mCherry-Mcl-1 reveals longer fluorescence lifetime of GFP-Bok(L70E)ΔTM as compared to GFP-Bok indicating reduced/lost interaction of GFP-Bok(L70E)ΔTM with mCherry-Mcl-1 as compared to GFP-Bok. B) Direct comparison of fluorescence lifetime of GFP-Bok alone with that of GFP-Bok and GFP-BokΔTM in the presence of mCherry-Mcl-1 shows reduced lifetime in the presence but not in the absence of the Bok TM-domain. C) Fluorescence lifetime of GFP-Bok is reduced by co-expression of mCherry-Mcl-1. The fluorescence lifetime of GFP-Bok(L70E)ΔTM is not reduced by co-expression of mCherry-Mcl-1. Also, co-expression of mCherry-Mcl-1ΔTM does not induce a reduction in the fluorescence lifetime of GFP-Bok.

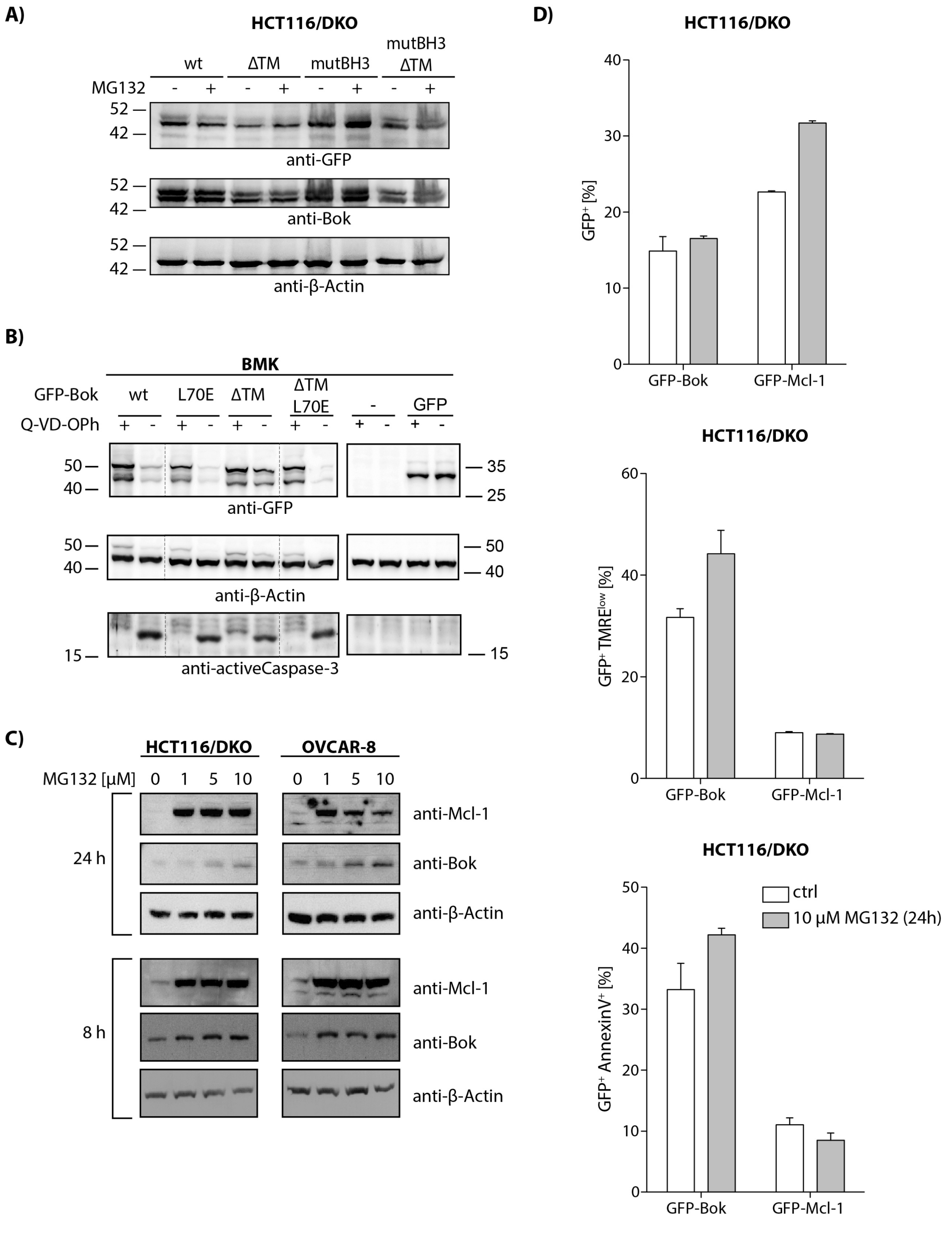

Supplemental Figure S3: A) Anti-GFP and anti-Bok Western blot analyses of HCT116/DKO cells transfected with vectors for the expression of GFP-Bok, GFP-BokΔTM, GFP-Bok(L70E) or GFP-Bok(L70E)ΔTM cultured in the presence or absence of 10 µM MG132 shows only slight stabilization of GFP-fused Bok-variants by MG132. B) Bax&Bak deficient BMK cells were transfected with expression vectors for the indicated GFP-Bok fusion proteins in the presence or absence of 10 µM Q-VD-OPh. Western blot analyses reveal that the pro-apoptotic activity of Bok-variants correlates with

detection of the active caspase-3 subunit. Inhibition of apoptosis by Q-VD-OPh results in enhanced expression levels of Bok variants which is most pronounced for highly pro-apoptotic forms like GFP-Bok(L70E)ΔTM and only weakly visible for GFP-BokΔTM. C) Western blot analysis of HCT116/DKO cells incubated in the presence or absence of various concentrations of MG132. Mcl-1 is significantly stabilized after 8 h and 24 h whereas the expression level of endogenous Bok is only slightly enhanced by MG132. D) Flow cytometric analysis of GFP-expression, PS-exposure and loss of $\Delta\Psi m$ in HCT116/DKO cells transfected with vectors for the expression of GFP-Bok or GFP-Mcl-1 reveals stabilization of GFP-Mcl-1 by MG132 whereas MG132 mediates only marginal enhanced number of GFP-Bok expressing cells (upper panel). In GFP-Bok transfected cells the number of $GFP^{+}/TMRE^{low}$ (middle panel) and GFP^{+}/Annexin V^{+} (lower panel) cells is considerably enhanced by MG132 while cell death is not enhanced in GFP-Mcl-1 expressing cells.

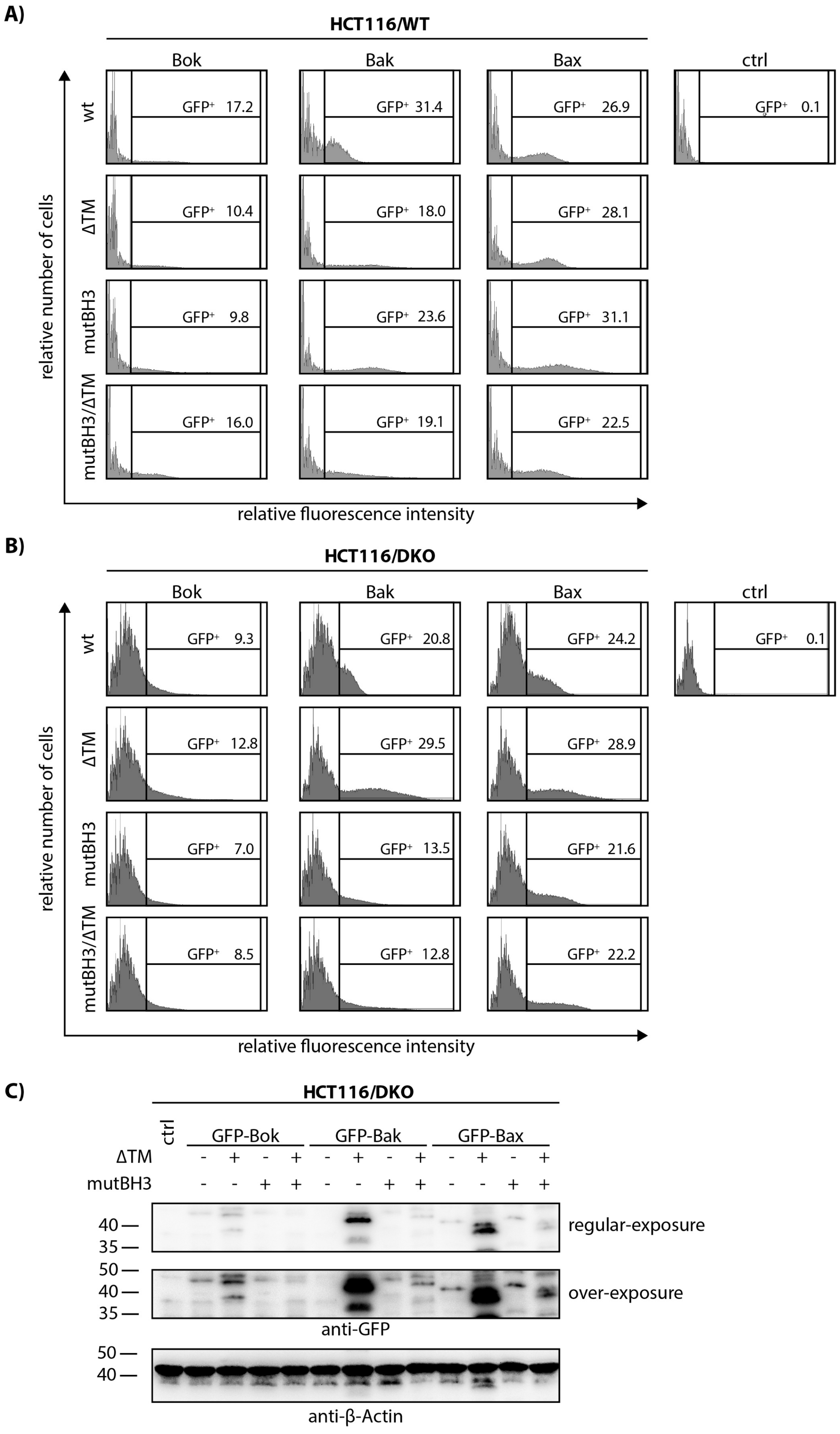
A)
HCT116/WT
Bok
Bak
Bax
ctrl
wt
ΔTM
mutBH3
mutBH3/ΔTM
relative number of cells
relative fluorescence intensity
GFP+ 17.2
GFP+ 31.4
GFP+ 26.9
GFP+ 0.1
GFP+ 10.4
GFP+ 18.0
GFP+ 28.1
GFP+ 9.8
GFP+ 23.6
GFP+ 31.1
GFP+ 16.0
GFP+ 19.1
GFP+ 22.5
B)
HCT116/DKO
GFP+ 9.3
GFP+ 20.8
GFP+ 24.2
GFP+ 0.1
GFP+ 12.8
GFP+ 29.5
GFP+ 28.9
GFP+ 7.0
GFP+ 13.5
GFP+ 21.6
GFP+ 8.5
GFP+ 12.8
GFP+ 22.2
C)
HCT116/DKO
ctrl
GFP-Bok
GFP-Bak
GFP-Bax
ΔTM
mutBH3
40 —
35 —
50 —
regular-exposure
over-exposure
anti-GFP
anti-β-Actin

Supplemental Figure S4: A and B) Histograms showing the gating of GFP$^+$ (%) HCT116/WT and HCT116/DKO cells after transfection with vectors for the expression of the indicated GFP-fusion proteins Bok (left), Bak (middle) or Bax (right) either WT (top) or with deleted transmembrane domain (ΔTM; 2nd row), mutation of the BH3-domain (mutBH3; 3rd row) or combination of both (mutBH3/ΔTM; bottom). C) Anti-GFP Western blot analysis of HCT116/DKO shown in B. Band intensity reflects the overall result from transfection efficiency in combination with expression level in individual cells.
